# Preparation of the Mouth for Anesthesia

**Published:** 1903-05-15

**Authors:** 


### PREPARATION OF THE MOUTH FOR ANESTHESIA.

Dr. Friteau called attention to the precautions to be observed, especially by the dental surgeon, in connection with the administration of general anesthetics, such as chloroform or ether. He advises the dentist to consult the patient's physician as to the advisability of using a general anesthetic and to decide upon its administration only after the physician's consent. The dentist should see to it that the mouth is put in as an aseptic a condition as is reasonably possible. All deposits should be removed and the patient should be instructed to use the following mouth-wash.

℞—Chloral hydrate, - - - 5 gm.
Saturated chloroform water, - - 250 "
Boiled water, - - - - 250 "
M.

Sig.—Use as a mouth wash every day for four or five days previous to the date set for the anesthesia.

Or,

℞—Neutral hydrogen dioxid, - - 250 gm.
Boiled water, - - - - 250 "
M.

Sig.—(Same as the previous formula.)

Or,

℞—Carbolic acid crystals, - - 5 gm.
Glycerin, - - - - - 50 "
Oil of peppermint, - - - 5 dps.
Boiled water, - - - - 500 gm.
M.

Before beginning the anesthesia the dentist himself should irrigate the patient's mouth and pharynx with one of these solutions. This procedure he considers very important, in view of the fact that whenever the dentist has to resort to a general anesthetic a considerable number of extractions have to be performed and the wounds and lacerations resulting from the operation will heal more rapidly if the mucous membrane is in a healthy condition. The author referred to the preparation of the operator, of the instruments to be employed, and of the operating room, the temperature of which should be at least 70°F. He recommends the administration to the patient of forty grams of magnesium citrate the day previous to the operation.

He then called attention to the post-operative care of the patient, stating among other things that rapid healing of the alveolar tissue can be brought about by irrigation with the chloral solution already given.—Prof. Friteau, in *Cosmos*.

